# Colchicine-like β-acetamidoketones as inhibitors of microtubule polymerization: Design, synthesis and biological evaluation of *in vitro* anticancer activity

**DOI:** 10.22038/ijbms.2019.34760.8242

**Published:** 2019-10

**Authors:** Ehsan Karimikia, Javad Behravan, Afshin Zarghi, Morteza Ghandadi, Sina Omid Malayeri, Razieh Ghodsi

**Affiliations:** 1 Biotechnology Research Center, Pharmaceutical Technology Institute, Mashhad University of Medical Sciences, Mashhad, Iran; 2 Department of Medicinal Chemistry, School of Pharmacy, Mashhad University of Medical Sciences, Mashhad, Iran; 3 Department of Pharmaceutical Chemistry, School of Pharmacy, Shahid Beheshti University of Medical Sciences, Tehran, Iran

**Keywords:** Anticancer activity, Chalcone, Colchicine, Colchicine–like, Molecular docking, Tubulin polymerization, β-acetamidoketones

## Abstract

**Objective(s):**

In this study a series of novel colchicine-like β-acetamidoketones was designed and synthesized as potential tubulin inhibitors

**Materials and Methods:**

The cytotoxicity of the novel synthesized β-acetamidoketones was assessed against two cancerous cell lines including MCF-7 (human breast cancer cells) and A549 (adenocarcinomic human alveolar basal epithelial cells) employing the MTT test. Tubulin polymerization test was done by using a commercial kit (tubulin polymerization assay kit).

**Results:**

In general, the cytotoxicity activities were highly dependent on the aromatic substitution pattern of phenyl ring at β position of β-acetamidoketones. Based upon, compound **4f **possessing the same structural elements of colchicine and chalcone 1, revealed the most cytotoxicity more than the other β-acetamidoketone against the cancerous cell lines and showed moderate antitubulin effect. The tubulin inhibitory effect of **4f**, colchicine and chalcone 1 were consistent with their antiproliferative activities. Molecular docking studies of **4f**, into the colchicine-binding site of tubulin exhibited possible mode of interaction between this compound and tubulin.

**Conclusion:**

The structure activity relationship (SAR) data attained showed that the presence of trimethoxy phenyl attached to carbonyl group of β-acetamidoketones and a methoxy group at *para* position of the other ring are essential for cytotoxic activity. In general, the cytotoxicity activities were highly dependent on the aromatic substitution pattern of phenyl ring at β position of β-acetamidoketones.

## Introduction

Cancer is the reason of one-quarter of all expiries in industrialized countries. Nowadays cancer is the second reason of death in the United States, and is predicted to exceed heart illnesses as the leading cause of death in the next years (1). Therefore; the discovery of anticancer, non-toxic and drug-like active compounds is still an urgent demand.

**Figure 1:**
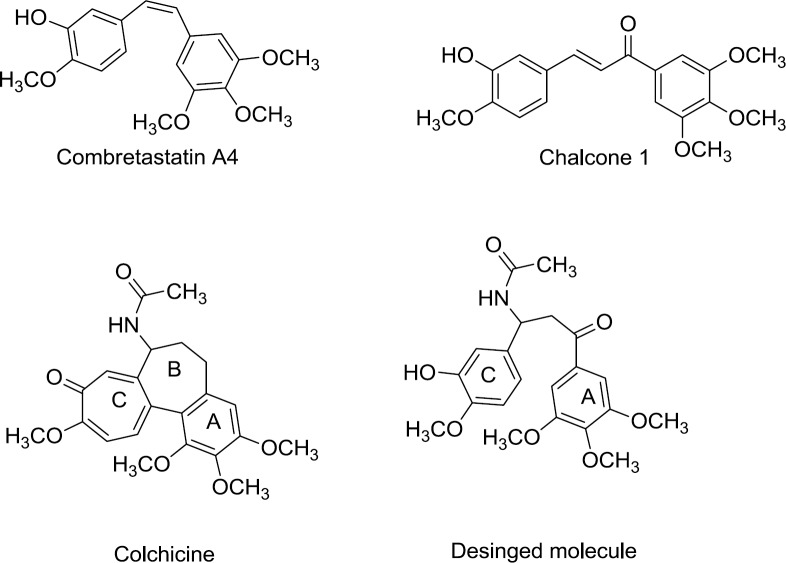
Chemical structures of known tubulin inhibitors (lead compounds) and designed compound

Microtubules play key role in mitosis and are imperative target for the design of novel anticancer agents. Colchicine (Figure 1) a compound isolated from *Colchicum autumnale* and *Gloriosa superba* was the first drug identified to bind tubulin, and it binds at a specific site called the colchicine domain (2). There are various compounds discovered, which bind tubulins at the colchicine binding site, and hence by inhibiting microtubule polymerization cause cell cycle arrest and, lead to cell death. (3). Combretastatins, separated from the South African tree *Combretum caffrum,* are a series of antimitotic agents. Combretastatin A-4 (CA-4, Figure 1) is a natural anti-tubulin compound which binds to colchicine’s binding site of tubulin and exerts its antimitotic effect. Many structural modifications of the combretastatin A-4 molecule, such as variation of substitutions on the A- and B-rings, have been reported (4-6). Numerous Chalcones are also reported as tubulin inhibitors (7, 8). Chalcone 1, a combretastatin-like derivative was known to possess potent anticancer activity and tubulin inhibition effect (7, 9).

Colchicine is identified to have significant anti-mitotic, anti-inflammatory and anti-fibrotic effects. Although colchicine possesses notable *in vitro* antitumor effects, its therapeutic uses have been restricted because of the low bioavailability and high toxicity (10). Though it’s unfavorable pharmacological profiles, colchicine is still a lead compound for the discovering of possible antimitotic drugs. Thus, various analogs of colchicine (11-14) have been synthesized with the goal of discovering novel, valuable drugs with more bioavailability and favorable pharmacological effects. Structure-activity studies (15, 16) suggested that the trimethoxy phenyl ring (A) and the methoxy tropone ring (C) of colchicine include the minimal structural features of the compound needed for its binding to tubulin. On the other hand, a study (17) revealed that the B-ring of colchicine plays a key role in the stability of tubulin binding while the A and the C-rings have not significant effect on it and colchicine binds at the ᾳ/β interface of tubulin. The B-ring binds on the ᾳ-subunit and the A and the C-rings bind on the β-subunit.

In the present study we report the design and synthesis of novel β-acetamidoketones possessing pharmacophoric requirements of anti-tubulins with central β-acetamido ketone bridge (Figure 1). They designed to bear some similarity to colchicine and chalcones. We designed the hybrid compounds, combining ring A and a part of ring B of colchicine and a fragment of chalcone 1 with the same linker length. (Figure 1). They were evaluated for their antiproliferative properties against two human cancer cell lines including MCF-7 and A549. The tubulin inhibitory effect of **4f** (the most cytotoxic compound) was also evaluated. In order to get the better structure activity relationship (SAR) data, we also evaluated the cytotoxicity and tubulin inhibitory properties of chalcone 1.

***Experimental***

All reagents, chemicals and solvents used in the present study were bought from Merck AG and Aldrich Chemical. Melting points were measured using a Thomas-Hoover capillary apparatus. Infrared spectra were attained by a Perkin Elmer Model 1420 spectrometer (Germany). ^1^HNMR and ^13^CNMR spectra were attained by Bruker FT-300 MHz instrument (Brucker Biosciences, USA). Chloroform-D was used as solvent. Coupling constant (J) values were assessed in hertz (Hz) and spin multiples were given as s (singlet), d (double), t (triplet), q (quartet), m (multiplet). The mass spectra were acquired using a 6410 Agilent LCMS triple quadrupole mass spectrometer (LCMS) with an electrospray ionization (ESI) interface (Japan). Elemental analyses were done on a Cos-Tec model EAS 4010 instrument (Cernusco, Italy) and the results were within ±0.4% of the theoretical values. 

***General procedure for the synthesis of β-acetamido propiophenones***

Substituted benzaldehyde (1.0 mmol) and acetyl chloride (0.5 ml) in acetonitrile (3.0 ml), TFA (0.30 mole %) were added to a solution of an appropriate acetophenone (1.0 mmol), and the mixture was stirred for about 30 min at an ice-water bath, and then permitted to warm to room temperature. upon completion the reaction, the mixture was transferred into ice-water (20.0 ml) saturated NaHCO_3_ was added to the mixture to adjust the pH to 7, which resulted to precipitation of the target β-acetamidoketone. The precipitate was filtered, washed with hexane and recrystallized in methanol. 

***β*** ***-*** ***acetamido*** ***-*** ***β*** ***-(*** ***phenyl)-3,4,5-trimethoxy propio-phenone (4a*** ***)***

Yield, 69%; mp= 101-103 ^°^C: IR (KBr) ν (cm^-1^): 1658 (C=O), 3266 (NH); ^1^HNMR (300 MHz) (CDCl_3_) δ (ppm): 1.67 (s, 3H, CH_3_), 3.35-3.43(dd, 1H, CH_2_, J=6.6 & 16.2 Hz), 3.78-3.85 (dd, 1H, CH_2_, J=6.6 & 16.2 Hz), 3.88 (s, 6H, OCH_3_), 3.90 (s, 3H, OCH_3_), 5.53-5.59 (m, 1H, CH), 6.61-6.63 (d, 1H, NH, J=7.5), 7.21 (s, 2H, 3,4,5-trimethoxyphenyl H_2 _& H_6_), 7.25-7.36 (m, 5H, phenyl):^ 13^C NMR (75 MHz) (CDCl_3_) δ (ppm): 23.38, 43.34, 50.48, 56.29, 60.94, 105.69, 126.58, 127.57, 128.71, 131.79, 140.78, 142.85, 153.07, 169.57, 197.14; MS (ESI) m/z:358.2 [M+1], 380.2 [M+23]. ). Anal. Calcd for C_20_H_23_NO_5_: C, 67.21; H, 6.49; N, 3.92. Found: C, 67.43; H, 6.31; N, 4.12.

**Scheme 1:**
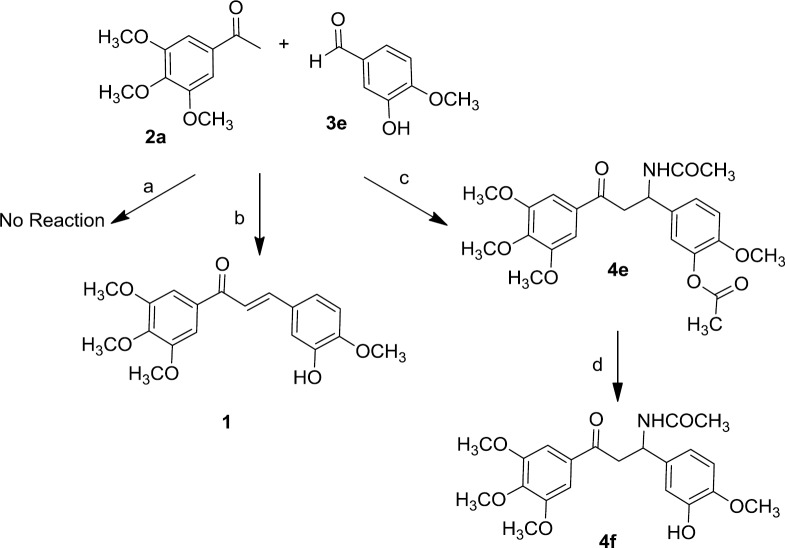
Reagents and conditions: (a) Cerium (IV) sulfate, CH_3_CN (b) H_3_BO_3_, CH_3_CN, CH_3_COCl (c) TFA, CH_3_CN, CH_3_COCl (d) K_2_CO_3_, NMP

**Scheme 2:**
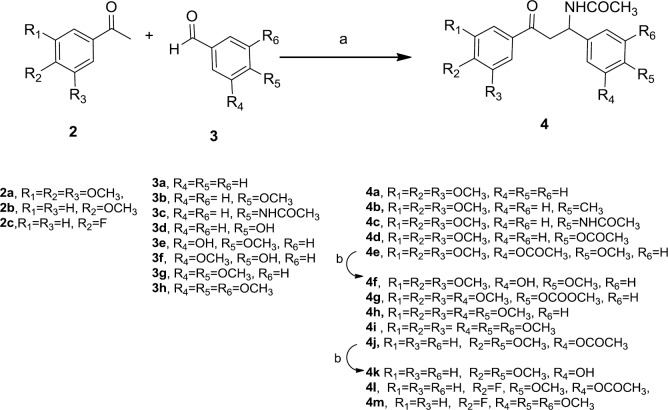
Reagents and conditions: (a) TFA, CH_3_CN, CH_3_COCl (b) K_2_CO_3_, NMP, 100 ^°^C

**Figure 2:**
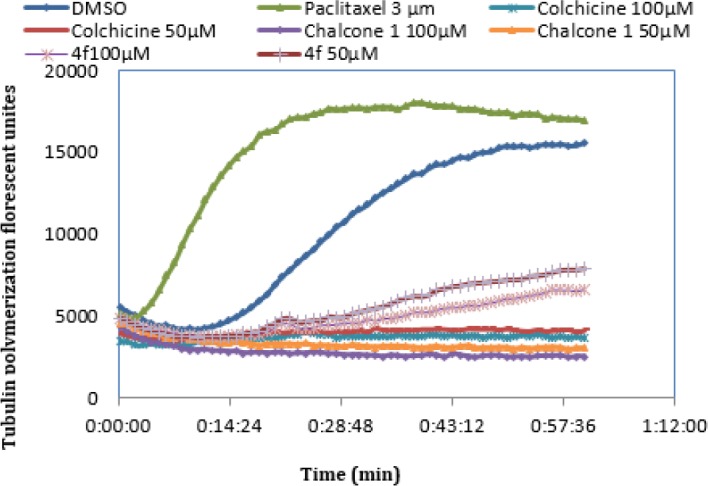
Effect of compounds (**4f**, Colchicine, Chalcone 1 and Paclitaxel) on *in vitro* tubulin polymerization

**Figure 3:**
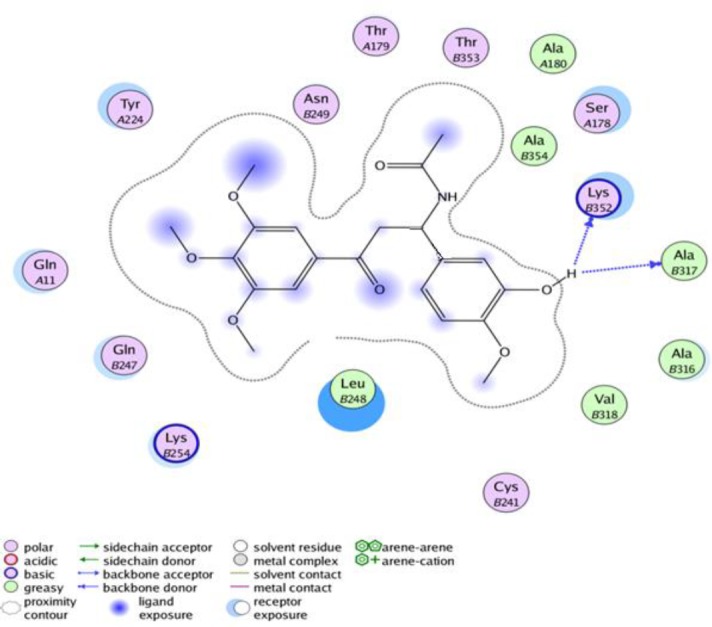
The interaction of compound **4f** in the crystal assembly of tubulin

**Table 1:** The* in vitro* anti-proliferative activities of colchicine-like β-acetamidoketones, colchicine, and chalcone 1 against human cancer cell lines

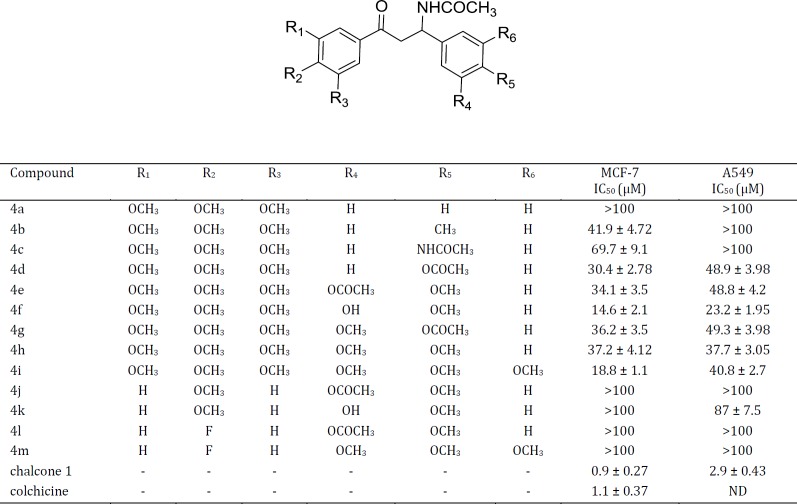

***β*** ***-*** ***acetamido*** ***-*** ***β*** ***-(4-*** ***methyphenyl)-3,4,5-trimethoxy propiophenone (4b*** ***)***

Yield, 64%; mp= 118-120°C: IR (KBr) ν (cm^-1^): 1647 (C=O), 3319 (NH) ):^ 1^HNMR (300 MHz) (CDCl_3_) δ (ppm): 2.05 (s, 3H, CH_3_), 2.33 (s, 3H, CH_3_), 3.32-3.3.40 (dd, 1H, CH_2_, J=6.9 & 16.2 Hz), 3.77-3.84 (dd, 1H, CH_2_, J=6.9 &16.2 Hz), 3.77 (s, 6H, OCH_3_), 3.79 (s, 3H, OCH_3_), 5.50-5.52 (m, 1H, CH), 6.51-6.53 (d, 1H, NH, J=7.5), 7.14-7.16 (d, 2H, 4-methylphenyl H_3_ &H_5_, J=7.8), 7.22-7.25 (m, 4H, 3,4,5-trimethoxyphenyl H_2 _& H_6 _& 4-methylphenyl H_2 _& H_6_):^ 13^CNMR (75 MHz) (CDCl_3_) δ (ppm): 21.05, 23.46, 43.37, 50.39, 56.30, 60, 95, 105.72, 126.50, 129.42, 131.81, 137.31, 137.64, 142.83, 153.08, 169.48, 197.26; MS (ESI) m/z: 372.2 [M+1], 394.2 [M+23]. Anal. Calcd for C_21_H_25_NO_5_: C, 67.91; H, 6.78; N, 3.77. Found: C, 67.63; H, 6.41; N, 3.42.

***β*** ***-*** ***acetamido*** ***-*** ***β*** ***-(4-*** ***acetamidophenyl)-3,4,5-trime-thoxy propiophenone (4c*** ***)***

Yield, 69%; mp= 155-157°C: IR (KBr) ν (cm^-1^): 1654 (C=O), 3061, 3288 (NH); ^1^HNMR (300 MHz) (CDCl_3_) δ (ppm): 2.06 (s, 3H, CH_3_), 2.18 (s, 3H, CH_3_), 3.32-3.40 (dd, 1H, CH_2_, J=6.6 &16.5 Hz), 3.75-3.82(dd, 1H, CH_2_, J=6.6 &16.5 Hz), 3.92 (s, 6H, OCH_3_), 3.94 (s, 3H, OCH_3_), 5.51-5.55 (dd, 1H, CH, J=6.9 & 12.3 Hz), 6.58-6.61 (d, 1H, NH, J=7.8), 7.20(s, 2H, 3,4,5-trimethoxyphenyl H_2 _& H_6_), 7.29-7.31 (d, 2H, 4-acetamidophenyl H_3_ &H_5_, J=8.4), 7.44-7.47 (d, 2H, 4-acetamido phenyl H_2 _& H_6_, J=8.4);^ 13^CNMR (75 MHz) (CDCl_3_) δ (ppm): 27.86, 28.93, 49.09, 54.79, 61.03, 65.52, 110.45, 124.64, 132,01, 136.75, 141.25, 143.01, 147.17, 157.75, 173.60, 174.34, 201.13: MS (ESI) m/z: 415.2 [M+1], 437.2 [M+23]. Anal. Calcd for C_22_H_26_N_2_O_6_: C, 67.91; H, 6.78; N, 3.77. Found: C, 67.55; H, 6.91; N, 3.27.

***β*** ***-*** ***acetamido*** ***-*** ***β*** ***-(4-*** ***acetoxyphenyl)-3,4,5-trimethoxy propiophenone (4d*** ***)***

Yield, 55%; mp= 137-140 ^°^C: IR (KBr) ν (cm^-1^): 1668, 1751 (C=O), 3284 (NH); ^1^HNMR (300 MHz) (CDCl_3_) δ (ppm): 2.00 (s, 3H, CH_3_), 2.28 (s, 3H, CH_3_), 3.32-.3.39 (dd, 1H, CH_2_, J=6.6 &16.5 Hz), 3.70-3.77 (dd, 1H, CH_2_, J=6.6 &16.5 Hz), 3.90 (s, 6H, OCH_3_), 3.91 (s, 3H, OCH_3_), 5.49-5.55 (m, 1H, CH), 6.75-6.78 (d, 1H, NH, J=7.5), 7.19 (s, 2H, 3,4,5-trimethoxyphenyl H_2_&H_6_), 7.01-7.04 (d, 2H, 4-acetoxyphenyl H_3_ &H_5_, J=8.4), 7.33-7.36 (d, 2H, 4-acetoxyphenyl H_2_&H_6_, J=8.4);^ 13^CNMR (75 MHz) (CDCl_3_) δ (ppm): 21.11, 23.35, 43.20, 49.91, 56.30, 60.95, 105.67, 121.78, 127.82, 131.67, 138.39, 142.92, 149.90, 153.10, 169.49, 169.65, 196.95: MS (ESI) m/z: 416.2 [M+1], 438.2 [M+23]. Anal. Calcd for C_22_H_25_NO_7_: C, 67.91; H, 6.78; N, 3.77. Found: C, 68.15; H, 6.41; N, 3.67.

***β*** ***-*** ***acetamido*** ***-*** ***β*** ***-(3-*** ***acetoxy, 4-methoxyphenyl)-3,4,5-trimethoxy propiophenone (4e*** ***)***

Yield, 56%; mp= 183-185 ^°^C: IR (KBr) ν (cm^-1^): 1618, 1664, 1758 (C=O), 3240 (NH); ^1^HNMR (300 MHz) (CDCl_3_) δ (ppm): 2.03 (s, 3H, CH_3_), 2.31 (s, 3H, CH3), 3.33-3.39 (dd, 1H, CH_2_, J=6.9 & 16.5 Hz), 3.76-3.81(m, 4H, CH_2_& OCH_3_), 3.82-3.85 (m, 9H, OCH_3_), 5.46-5.51 (m, 1H, CH), 6.50-6.52 (d, 1H, NH, J=7.6 Hz), 6.91-6.93 (d, 1H, 3-acetoxy-4-methoxyphenylH_5_, J=8.4 Hz), 7.05-7.06 (d, 1H,3-acetoxy-4-methoxyphenyl H_2_, J=2.4 Hz), 7.18-7.20 (dd, 1H, 3-acetoxy-4-methoxyphenyl H_6_, J= 2.4&8.4Hz), 7.22(s, 2H, 3,4,5-trimethoxyphenyl H_2 _& H_6_); ^13^CNMR (75 MHz) (CDCl_3_) δ (ppm): 20.64, 23.32, 43.21, 49.73, 55.91, 56.29, 60.92, 105.68, 105.80, 112.36, 121.38, 125.21, 131.71, 133.31, 139.70, 142.81, 150.42, 153.07, 168.94, 169.63, 196.95:MS (ESI) m/z:446.2 [M+1], 468.2 [M+23]. Anal. Calcd for C_23_H_27_NO_8_: C, 67.91; H, 6.78; N, 3.77. Found: C, 68.25; H, 6.81; N, 3.68

***β*** ***-*** ***acetamido*** ***-*** ***β*** ***-(4-*** ***acetoxy, 3-methoxyphenyl)-3,4,5-trimethoxy propiophenone (4g*** ***)***

Yield, 67%; mp= 135-137 ^°^C: IR (KBr) ν (cm^-1^): 1617,1774 (C=O), 3240(NH); ^1^H NMR (300 MHz) (CDCl_3_) δ (ppm): 2.01 (s, 3H, CH3), 2.28 (s, 3H, CH3), 3.31-3.35 (dd, 1H, CH_2_, J=6.9 &16.41 Hz), 3.74-3.79 (dd, 1H, CH_2_, J=6.9 &16.41 Hz), 3.80 (s, 3H, OCH_3_), 3.88-4.04 (m, 9H, OCH_3_), 5.44-5.48(m, 1H, CH), 6.57 (s, 1H, NH), 6.88-6.90(d, 1H,4-acetoxy-3-methoxyphenyl H_5_, J=8.51), 7.02-7.03(d, 1H,4-acetoxy-3-methoxyphenyl H_2_, J=2.12Hz), 7.15-7.17 (dd, 1H,4-acetoxy-3-methoxyphenyl H_6_, J= 2.1 &8.51Hz), 7.20 (s, 2H, 3,4,5-trimethoxyphenyl H_2_&H_6_);^ 13^CNMR (75 MHz) (CDCl_3_) δ (ppm): 20.66, 23.43, 43.08, 49.78, 55.94, 56.33, 60.95, 105.72, 112.40, 121.40, 125.21, 131.71, 133.24, 139.75, 142.92, 150.48, 153.12, 168.93, 169.50, 197.05:MS (ESI) m/z: 446.2 [M+1], 468.2 [M+23]. Anal. Calcd for C_23_H_27_NO_8_: C, 67.91; H, 6.78; N, 3.77. Found: C, 68.29; H, 6.86; N, 3.88.

***β*** ***-*** ***acetamido*** ***-*** ***β*** ***-(3,4-*** ***dimethoxyphenyl)-3,4,5-trimethoxy propiophenone (4h*** ***)***

Yield, 81%; mp= 125-127 C: IR (KBr) ν (cm^-1^): 1653 (C=O), 3316(NH); ^1^H NMR (400 MHz) (CDCl_3_) δ (ppm): 2.06 (s, 3H, CH3), 3.31-3.38(dd, 1H, CH_2_, J=7.2 & 16.2 Hz), 3.77-3.84(dd, 1H, CH_2_, J=7.2 & 16.2 Hz), 3.86 (s, 3H, OCH_3_), 3.88 (s, 3H, OCH_3_), 3.93 (s, 6H, OCH_3_), 3.94 (s, 3H, OCH_3_), 5.47-5.50(m, 1H, CH), 6.48-6.50 (d, 1H, NH, J=7.8), 6.81-6.90(m, 3H,3, 4-dimethoxyphenyl, H_2_, H_5_&H_6_) 7.23(s, 2H, 3,4,5-trimethoxyphenyl, H_2_&H_6_):^ 13^C NMR (75 MHz) (CDCl_3_) δ (ppm): 23.47, 43.36, 50.59, 55.88, 55.92, 56.31, 60.95, 105.74, 110.44, 111.20, 118.58, 131.81, 133.17, 142.88, 148.49, 149.05, 153.10, 169.46, 197.33; MS (ESI) m/z:418.2 [M+1], 440.2 [M+23]. Anal. Calcd for C_22_H_27_NO_7_: C, 67.91; H, 6.78; N, 3.77. Found: C, 67.59; H, 6.86; N, 3.98.

***β*** ***-*** ***acetamido*** ***-*** ***β*** ***-(3,4,5-*** ***trimethoxyphenyl)-3,4,5-trimethoxy propiophenone (4i*** ***)***

Yield, 71%; mp= 133-135 ^°^C: IR (KBr) ν (cm^-1^): 1659 (C=O), 3246(NH); ^1^H NMR (300 MHz) (CDCl_3_) δ (ppm):1.74 (s, 3H, CH3),3.28-3.35 (dd, 1H, CH_2_, J=6.9&15.9 Hz), 3.72-3.79 (dd, 1H, CH_2_, J=6.9&15.9 Hz), 3.81 (s, 3H, OCH_3_), 3.83(s, 6H, OCH_3_), 3.92 (s, 6H, OCH_3_), 3.95(s, 3H, OCH_3_), 5.44-5.46(m, 1H, CH), 6.55(s, 2H, 3,4,5-trimethoxyphenyl H_2_&H_6_), 6.60-6.62 (d, 1H, NH, J=7.5), 7.20(s, 2H, 3,4,5-trimethoxyphenyl H_2_&H_6_);^ 13^C NMR (75 MHz) (CDCl_3_) δ (ppm): 23.36, 43.40, 51.01, 56.09, 56.29, 60.73, 60.93, 103.88, 105.71, 131.82, 136.53, 137.29, 142.86, 153.08, 153.29, 169.54, 197.22; MS (ESI) m/z:448.2 [M+1], 470.2 [M+23]. Anal. Calcd for C_23_H_29_NO_8_: C, 61.73; H, 6.53; N, 3.13. Found: C, 62.19; H, 6.76; N, 3.26.

***β*** ***-*** ***acetamido*** ***-*** ***β*** ***-(3-*** ***acetoxy, 4-methoxyphenyl)-4-methoxy propiophenone (4j*** ***)***

Yield, 65%; mp= 156-157°C: IR (KBr) ν (cm^-1^): 1648, 1691, 1765 (C=O), 3281(NH); ^1^H NMR (300 MHz) (CDCl_3_) δ (ppm): 2.01 (s, 3H, CH_3_), 2.30 (s, 3H, CH_3_), 3.37-3.43 (dd, 1H, CH_2_, J=6.4 &16.4 Hz), 3.66-3.72 (dd, 1H, CH_2_, J=6.4 &16.4 Hz), 3.80 (s, 3H, OCH_3_), 5.50-5.55(m, 1H, CH), 6.76-6.77 (d, 1H, NH, J=7.5 Hz ), 6.88-6.90(d, 1H, 3-acetoxy-4-methoxyphenyl H_5, _J= 8.4 Hz), 6.92-6.94 (d, 2H, 4-methoxyphenyl H_3 _& H_5_, J= 8.8 Hz), 7.05(d, 1H, 3-acetoxy-4-methoxyphenyl H_2, _J= 2 Hz), 7.16-7.18 (dd, 1H, 3-acetoxy-4-methoxyphenyl H_6, _J= 2 & 8.4 Hz) 7.90-7.92 (m, 2H, 2H, 4-methoxyphenyl H_2_&H_6_, J= 8.8 Hz);^ 13^CNMR (75 MHz) (CDCl_3_) δ (ppm): 20.65, 23.40, 42.73, 49.33, 55.51, 55.93, 111.82, 112.33, 113.87, 121.29, 125.04, 129.70, 130.52, 133.88, 139.70, 150.29, 163.83, 169.45, 196.93; MS (ESI) m/z:408.2 [M+23]. Anal. Calcd for C_21_H_23_NO_6_: C, 65.44; H, 6.02; N, 3.63. Found: C, 65.56; H, 6.36; N, 3.39.

***β*** ***-*** ***acetamido*** ***-*** ***β*** ***-(3-*** ***acetoxy, 4-methoxyphenyl)-4-fluoro propiophenone (4l*** ***)***

Yield, 89%; mp= 155-157°C: IR (KBr) ν (cm^-1^): 1648, 1691, 1765 (C=O), 3281(NH); ^1^H NMR (300 MHz) (CDCl_3_) δ (ppm): 2.01 (s, 3H, CH3), 2.30 (s, 3H, CH_3_), 3.33-3.39 (dd, 1H, CH_2_, J=6.0 & 16.4 Hz), 3.64-3.69 (dd, 1H, CH_2_, J=6.0 &16.4 Hz), 3.80 (s, 3H, OCH_3_), 5.48-5.52 (m,1H, CH), 6.75-6.77 (d, 1H, NH, J=7.5Hz), 6.88-6.90 (dd, 1H, 3-acetoxy-4-methoxyphenyl, H_6_, J= 2 & 8 Hz), 6.95-6.98 (m, 2H, 3-acetoxy-4-methoxyphenyl H_2_&H_5_), 7.10-7.17 (m, 2H, 4-flouroophenyl, H_2_&H_6_), 7.93-7.95 (m, 2H, 4-flourophenyl, H_3 _& H_5_); MS (ESI) m/z: 374.1 [M+1], 396.1 [M+23]. Anal. Calcd for C_20_H_20_FNO_5_: C, 64.34; H, 5.40; N, 3.75. Found: C, 64.76; H, 6.27; N, 3.84.

***β*** ***-*** ***acetamido*** ***-*** ***β*** ***-(3,4,5-*** ***trimethoxyphenyl)- 4-fluoro propiophenone (4m*** ***)***

Yield, 85%; mp= 186-188 ^°^C: IR (KBr) ν (cm^-1^): 1618, 1690 (C=O), 3242 (NH); ^1^HNMR (300 MHz) (CDCl_3_) δ (ppm): 2.08 (s, 3H, CH3), 3.35-3.41 (dd, 1H, CH_2_, J=6.4 &16.4 Hz), 3.69-3.74 (dd, 1H, CH_2_, J=6.4 &16.4 Hz), 3.81 (s, 3H, OCH_3_), 3.83 (s, 6H, OCH_3_), 5.44-5.49 (m, 1H, CH) 6.55 (s, 2H, 3,4,5-trimethoxyphenyl H_2 _& H_6_), 6.67-6.69(d, 1H, NH, J=8Hz), 7.12-7.17(t, 2H, 4-flouroophenyl H_2 _& H_6_, J=8.8Hz), 7.95-7.98 (m, 2H, 4-flourophenyl H_3 _& H_5_):^ 13^CNMR (75 MHz) (CDCl_3_) δ (ppm): 23.40, 43.33, 50.63, 56.14, 60.77, 103.88, 115.74, 116.03, 130.80, 130.93, 133.11, 136.57, 153.34, 169.62, 197.04: MS (ESI) m/z: 376.2[M+1], 398.1 [M+23]. 

***General procedure for deacetylation of acetoxy β-acetamido ketones***

A mixture of acetoxy β-acetamido ketones (2.5 mmol) and K_2_CO_3_ (4 mmol) in NMP (2.5 ml) were heated at 100°C. After completion of the reaction (monitored by TLC), permitted to warm to room temperature and diluted with 2% aqueous NaOH (10 ml) and extracted with diethyl ether. After acidifying the aqueous layer with HCl (6 M) and extracting with diethyl ether, the ethereal phase was dehydrated using Na_2_SO_4,_ and vaporized under vacuum to obtain the target hydroxylated β-acetamido ketones. 

***β*** ***-*** ***acetamido*** ***-*** ***β*** ***-(3-*** ***hydroxy, 4-methoxyphenyl)-3,4,5-trimethoxy propiophenone (4f*** ***)***

Yield, 82%; mp= 123-125 ^°^C: IR (KBr) ν (cm^-1^): 1659 (C=O), 3303 (NH, OH); ^1^HNMR (300 MHz) (CDCl_3_) δ (ppm): 2.05 (s, 3H, CH_3_), 3.28-3.36 (dd, 1H, CH_2_, J=6.9 & 15.9 Hz), 3.74-3.81(dd, 1H, CH_2_, J=6.9 & 15.9 Hz), 3.87 (s, 3H, OCH_3_), 3.88-4.01 (m, 9H, OCH_3_), 5.45-5.46 (m, 1H, CH), 5.73 (s, 1H, OH), 6.51-6.53 (d, 1H, NH, J=6.9), 6.80-6.92(m, 3H,3-hydroxy-4-methoxyphenyl H_2_, H_5_&H_6_) 7.22 (s, 2H, 3,4,5-trimethoxyphenyl H_2 _& H_6_); MS (ESI) m/z:404.2 [M+1], 426.2 [M+23]. Anal. Calcd for C_21_H_25_NO_7_: C, 62.52; H, 6.25; N, 3.47. Found: C, 62.87; H, 6.44; N, 3.73.

***β*** ***-*** ***acetamido*** ***-*** ***β*** ***-(3-*** ***hydroxy, 4-methoxyphenyl)-4-methoxy propiophenone (4k*** ***)***

Yield, 84% ;mp= 120-122 ^°^C: IR (KBr) ν (cm^-1^): 1664 (C=O), 3357(NH, OH); ^1^H NMR (300 MHz) (CDCl_3_) δ (ppm): 2.03 (s, 3H, CH_3_),3.30-3.38 (dd, 1H, CH_2_, J=6.0&16.5 Hz), 3.63-3.70 (dd, 1H, CH_2_, J=6.0&16.5 Hz), 3.85 (s, 3H, OCH_3_), 3.88 (s, 3H, OCH_3_), 5.45-5.48 (m, 1H, CH), 5.71 (s, 1H, NH), 6.72 (s, 1H, OH), 6.75-6.95(m, 5H,3-hydroxy-4-methoxyphenyl H_2_, H_5 _& H_6 _& 4-methoxyphenyl H_3 _& H_5_), 7.91-7.94 (d, 2H, 4-methoxyphenyl H_2 _& H_6_, J=9 Hz); MS (ESI) m/z:709.3 [2M+23]. Anal. Calcd for C_19_H_21_NO_5_: C, 66.46; H, 6.16; N, 4.08. Found: C, 66.88; H, 6.73; N, 3.97.

***3-(3-hydroxy-4-methoxyphenyl)-1-(3,4,5-trimethoxyphenyl)prop-2-en-1-one(Chalcone1)***

The same as reference (9): mp= 144–146 ^°^C; IR (KBr) ν (cm^-1^): 1675 (C=O), 3416( OH); ^1^HNMR (300 MHz) (CDCl_3_) δ (ppm): 3.93 (s, 3H, OCH_3_), 3.94 (s, 9H, OCH_3_),5.72 (s, 1H, OH), 6.87 (d, 1H, 3-hydroxy-4-methoxyphenyl H_5_ , J= 8.0 Hz), 7.13 (dd, 1H, 3-hydroxy-4-methoxyphenyl H_6_, J= 2.0, 8.0 Hz),7.25–7.30 (m, 3H, 3-hydroxy-4-methoxyphenyl H_2 _& 3,4,5-trimethoxyphenyl, H_2_&H_6_), 7.33-7.36 (d, 1H, CH, J= 15.5 Hz), 7.72-7.7536 (d, 1H, CH, J= 15.5 Hz): MS (ESI) m/z: 345.1 [M+1], 367.1 [M+23]. Anal. Calcd for C_19_H_20_O_6_: C, 66.27; H, 5.85. Found: C, 66.58; H, 5.94.

***Cytotoxicity assay***

*General procedure*

The MTT (3-[4,5-dimethylthiazol-2-yl]-2,5-diphenyl tetrazolium bromide) based assay was performed by seeding 5000 cancerous cells per 180 µl RPMI complete culture medium in each well of 96-well culture plates (18-22). After 24 hr, culture medium was substituted with medium having positive control colchicine and chalcone 1 as well as diverse concentrations of new synthesized compounds and RPMI as negative control. Then cells were incubated at 37 ^°^C in 5% CO_2_ incubator for 48 hr. Then 25 µl of MTT solution (4 mg ml^-1^) were added to each well and further incubated at 37 ^°^C for 3 hr, then, formazan crystals were dissolved in DMSO (100 µl) and plates were read using a plate reader (Synergy H4, USA) at 540 nm. This test was done in triplicate determination each time.

*Tubulin polymerization assay*

Tubulin polymerization test was done by using a commercial kit (Tubulin Polymerization Assay Kit (Porcine tubulin and Fluorescence based Kit, Cat No BK011P, Cytoskeleton, USA)), based on the manufacturer’s procedure (23-25). Tubulin protein was added to tubulin buffer (80 mM PIPES, 2 mM MgCl_2_, 0.5 mM EGTA, 1 mM GTP, 60% (v/v) glycerol, pH 6.9) then poured to wells of a 96-well plate possessing the cytotoxic chemicals or vehicle and then mixed well. The effects of compound **4f** and Chalcone 1 at 50 and 100 µM concentrations on tubulin polymerization were investigated.

Tubulin polymerization was monitored by detecting the enhancement of the fluorescence because of the addition of a fluorescence reporter into microtubules as polymerization occurs. Polymerization was measured by excitation at 360 nm and emission at 420 nm for 1 hr at 1 min breaks by a plate reader (Synergy H4, USA). Colchicine at 50 and 100 µM concentrations and paclitaxel at 3 µM concentration were used as positive destabilizing and stabilizing controls, respectively. 

*Molecular modeling*

Mode of interaction between **4f** and tubulin was evaluated by docking. 2D structure of the compound was organized in Chem Draw Ultera 8.0 software and 3D structure was prepared by Hyperchem 7 software via molecular mechanic force filed pre-optimization followed by AM1 semiempirical calculation. The X-ray crystal structure of tubulin (PDB ID: 1SA0) was copied from the Protein Data Bank (www.rcsb.org). Further changes such as polar hydrogen adding and water molecules deletion was done by MOE software. Compound **4f **was docked into the binding site of tubulin using MOE software. Every atom in a 5 Å about the co-crystallized ligand in crystal coordinates of tubulin was selected as binding site. The docking simulations were performed using triangle matcher placement algorithm in combination with London dG scoring function and force field as refinement process. The top-score docking poses were selected for final ligand–target interaction analysis using LigX module in MOE Software. Validation of docking method was first assessed by docking of co-crystalized ligand into the tubulin binding site (26).

## Results

***Synthesis***

As depicted in scheme 1, In order to synthesize **4f, **at first we let 3,4,5-trimethoxy acetophenone 2a and 3-hydroxy-4-methoxy benzaldehyde 3e condense in acetonitrile in the presence of catalytic amount of Cerium (IV) sulfate according to the reported procedure (27) but no reaction was happened. To get the target **4f,** we examined another reported method (28) employing boric acid as a catalyst and acetyl chloride in acetonitrile. Interestingly, the IR and ^1^H-NMR spectra revealed that the chalcone 1 was formed instead. Finally, we tried to do the reaction using trifloroacetic acid as a catalyst in the presence of acetyl chloride in acetonitrile (29). The presence of a peak in 1758 cm^-1^ in the IR spectrum and also a singlet peak in 2.31 ppm in ^1^HNMR spectrum proved the formation of 4e which possesses the acetoxy group instead of hydroxyl group. Hydrolysis of acetoxy group of 4e in the presence of potassium carbonate in NMP (N-methyl-2-pyrrolidone) at 100 ^°^C (28) led to the formation of **4f**. We found that TFA is the best catalyst for synthesis of our β-acetamidoketones (Scheme 2), and acetylation of hydroxyl group was observed while using substituted benzaldehyde bearing hydroxyl group (**3d**, **3e** and **3f**). The compounds were characterized by nuclear magnetic resonance, infrared, mass spectrometry and elementary analysis.

***Biological evaluation***

*In vitro anticancer activity*

The cytotoxicity of the synthesized compounds was assessed against two cancerous cell lines including MCF-7 (human breast cancer cells) and A549 (adenocarcinomic human alveolar basal epithelial cells) using the MTT test. As shown in Table 1, by comparing the cytotoxicity of **4j** and **4k** (which displayed no activity at the concentrations below 100 µM), with those of **4e** and **4f**, we can conclude that trimethoxy phenyl moiety attached to carbonyl group is essential for cytotoxic activity of these compounds. The cytotoxicity activity was highly dependent on the aromatic substitution pattern of phenyl ring at β position of β-acetamidoketones. SAR data showed that, introducing an electron donating group in the *para* position of **4a** increased its cytotoxic activity against MCF-7 cells (see the anti-proliferative activities of **4a**-**4d **in Table 1). According to our results, compound **4f** was at least twice more cytotoxic than acetoxy analogue **4e**. Comparing the cytotoxic activity of **4f** with its parent compound **4e** shows that the hydroxyl group at *meta* position of phenyl ring has a crucial role for its activity which may explain the ability of hydroxyl group to form hydrogen bond within the active site of tubulin as a hydrogen binding donor. Replacement of the acetamido group at *para* position of phenyl ring (**4c)** by acetoxy substituent (**4d)** increased cytotoxic activity considerably. Our results indicated that compound **4i** possessing trimethoxy phenyl ring at β position showed more cytotoxicity activities than **4h** having dimethoxy phenyl ring in MCF-7 cells. According to our results, compound **4f** possessing the colchicine and chalcone 1 aromatic substitution pattern showed the highest cytotoxicity among the β-acetamidoketones series against the cancer cell lines. As chalcone 1 showed higher anti-proliferative activity compared to colchicine, it can be concluded that ᾳ, β-unsaturated bridge of chalcone 1 might act as the Michael acceptor in tubulin binding site or other targets in addition to rigid linkage. In general, our compounds exhibited more cytotoxicity effects in MCF-7 cancer cells compared to A549 cancer cells, demonstrating that the compounds may exert their cytotoxicity with different mechanisms in different cancer cells.

***Tubulin polymerization assay***

In order to clarify whether the cytotoxic activity of **4f** was related to the tubulin binding ability, compounds **4f **(the most cytotoxic compound), chalcone 1 and reference compound colchicine (polymerization suppressor) and a polymerization promoter (paclitaxel) were assessed for its effect on tubulin polymerization. As illustrated in Figure 2, **4f** proved to be inhibitor of tubulin polymerization in a manner similar to that of chalcone 1 and colchicine. However, **4f** was not as potent as these reference tubulin inhibitors but its tubulin inhibition effect was dose dependent. SAR acquired data indicated that flexible bridge in **4f**, could lead to conformational flexibility that is unfavorable for tubulin inhibition. Because of higher tubulin inhibitory effect of chalcone 1 compared with colchicine it can be concluded that ᾳ, β-unsaturated bridge of chalcone might act as the Michael acceptor in tubulin binding site in addition to the rigid linkage. The tubulin inhibitory effect of **4f**, colchicine and chalcone 1 were consistent with their antiproliferative activities. On the other hand, the order of tubulin inhibitory effect of these three compounds is the same as their antiproliferative activities.

***Molecular modeling (docking) studies***

In an attempt to elucidate the possible binding mode of this novel series of tubulin inhibitors, docking of the most potent compound **4f** was performed at the active site of the tubulin dimer. The quality and validity of docking procedure were investigated by docking of co-crystalized ligand within the binding site of tubulin. From docking studies, the top binding position exhibited a similar alignment in the binding pocket to the co-crystallized ligand found in crystal assembly (PDB ID of 1SA0). The root mean square deviation (RMSD) between co crystallized ligand within the binding site and ligand docked in the crystal structure of tubulin was 1.4 Å, indicative of a proper ability to mimic the ligand binding mode recognized in the experimental data (30, 31). As mentioned above, compound **4f** possessing structural elements of colchicine and chalcone 1, demonstrated the most anti proliferative activity compared to the other synthesized compounds which inhibited the polymerization of tubulin. According to ligand interaction mode of **4f** by LigX module of MOE software, hydroxyl group of **4f**, could form hydrogen bonds with LysB352 or AlaB317 (Figure 3). The methoxy groups of the first phenyl ring made contacts with the backbone of several amino acid residues such as TyrA224, GlyA11, GlnB247 and LysB254. This phenyl ring of **4f** provided additional interaction with LeuB 248. The other ring of **4f** made contacts with the aliphatic chain of residues CysB241, ValB318, AlaB316, AlaB317 and LysB352. The β-acetamido side chain of **4f **was surrounded by residues AlaB354, AlaA180, SerA178, ThrB353, ThrA179 and AsnB249 and could make contacts with them. These hydrophobic interactions and hydrogen bonds formation of **4f** with tubulin binding site can explain inhibitory effect of this compound. 

## Discussion

A new series of colchicine-like β-acetamidoketone analogues was synthesized and evaluated for their cytotoxic activity against MCF-7 and A549 cancer cells. The structure activity data acquired indicate that the presence of trimethoxy phenyl attached to carbonyl group of and a methoxy group at *para* position of the other ring in the synthesized compounds are essential for cytotoxic activity. As chalcone 1 showed higher anti-proliferative activity compared to colchicine, it can be concluded that ᾳ, β-unsaturated bridge of chalcone 1 might act as the Michael acceptor in tubulin binding site or other targets in addition to rigid linkage. In general, the cytotoxic activities were highly dependent on the aromatic substitution pattern of the phenyl ring at position β of β-acetamidoketones. Based upon, compound **4f** possessing the trimethoxyphenyl and 3-hydroxy-4-methoxy groups, demonstrated the best cytotoxicity among the other β-acetamidoketones against the cancerous cell lines and proved to be a tubulin inhibitor. Finally, molecular docking studies of **4f** into the colchicine-binding site of tubulin demonstrated the possible interactions of this compound at the active site of tubulin. The hydrophobic interactions and hydrogen bonds formation of **4f** with tubulin binding site can explain inhibitory effect of this compound.

## Conclusion

The cytotoxicity activities were highly dependent on the aromatic substitution pattern of phenyl ring at position β of β-acetamidoketones. Based upon, compound **4f** possessing the same structural elements of colchicine and chalcone 1, revealed the most cytotoxicity more than the other β-acetamidoketone against the cancerous cell lines and showed moderate antitubulin effect. The tubulin inhibitory effect of **4f**, colchicine and chalcone 1 were consistent with their antiproliferative activities. Molecular docking studies of **4f**, into the colchicine-binding site of tubulin exhibited possible mode of interaction between this compound and tubulin.
